# Unusual osseous presentation of blastomycosis in an immigrant child: a challenge for European pediatricians

**DOI:** 10.1186/1824-7288-38-69

**Published:** 2012-12-10

**Authors:** Margherita Codifava, Azzurra Guerra, Giulio Rossi, Paolo Paolucci, Lorenzo Iughetti

**Affiliations:** 1 Department of Pediatrics, University of Modena and Reggio Emilia, via del Pozzo 71, Modena, 41124, Italy; 2 Department of Pathology, AOU Policlinico of Modena, via del Pozzo 71, Modena, 41124, Italy

**Keywords:** Blastomyces dermatitidis, Blastomycosis, Osteomyelitis, Itraconazole

## Abstract

**Background:**

Blastomycosis, caused by the thermally dimorphic fungus Blastomyces dermatitidis is a systemic pyogranulomatous infection, endemic in United States and Canada, with few reported cases in Africa and Asia. It is uncommon among children and adolescents, ranging from 3% to 10%. Clinical features vary from asymptomatic spontaneously healing pneumonia, through acute or chronic pneumonia, to a malignant appearing lung mass. Blastomycosis can originate a "metastatic disease" in the skin, bones, genitourinary tract and central nervous system. Bone is the third most common site of blastomycotic lesions, after lung and skin. Bones may be involved in 14-60% of cases of blastomycosis. Direct visualization of single broadbased budding yeast with specific stains in sputum or tissue samples at microscopy is the primary method for diagnosis, while culture is timeconsuming and other methods are unreliable.

**Case presentation:**

We report a case of severe osteoarticular Blastomycosis occurring in a 3-years-old presented to our Emergency Department with pain and swelling of the left knee, successfully treated with surgical curettage and antifungal therapy. To our knowledge this is the first case reported in Europe.

**Conclusions:**

Blastomycosis represents a challenge for European physicians, and it should be included in the differential diagnosis of unexplained infections in patients coming from endemic areas.

## Background

Blastomycosis is a systemic pyogranulomatous disease caused by the thermally dimorphic fungus Blastomyces dermatitidis (Bd). It is endemic in Southern, Southeastern and Midwestern states of the United States and Canada, with few reported cases in Africa and Asia and no cases are reported in Europe. The organism’s ecological niche is wet soil that has been enriched with animal droppings, rotting wood and other decaying vegetable matter. Outdoor activities are associated with blastomycosis infection. Less commonly, direct cutaneous inoculation via a penetrating outdoor injury, a laboratory accident or an animal bite can occur. Disruption of wet soil or organic matter containing Bd mycelia releases infectious conidia, which are consequently inhaled by a susceptible host. In the lungs alveolar macrophages, neutrophils and monocytes provide natural resistance to infection with conidia. The clinical features of blastomycosis range from asymptomatic spontaneously healing pneumonia, through acute or chronic pneumonia, to a malignant appearing lung mass. If host responses in the lung fail to contain the infection, a lymphohematogenous spread follows, disseminating to almost any organ: skin, bones, genitourinary tract and central nervous system. Fulminant manifestation occurs in both immunocompetent and immunocompromised patients. Blastomycosis, unlike Aspergillosis or Candidiasis, is not considered an opportunistic infection, but AIDS or transplanted patients are more likely to have disseminated disease [1,2]. The ability to mimic other diseases often leads to erroneous diagnosis delaying the appropriate treatment. We describe a case of blastomycosis that occurred as a sporadic localized osteolytic lesion of the distal femur and caused a muscle abscess in a young African child. To our knowledge this is the first case described in Europe.

### Case presentation

**Figure 1:**
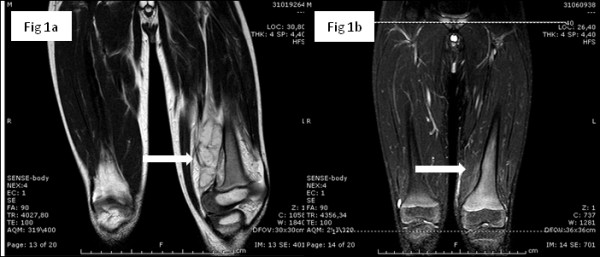
**MRI of the left knee prior and post curettage and antifungal therapy.** Figure 1a: voluminous neoformation (arrow) with expansive character, inhomogeneous signal intensity and polycyclic contours, extending longitudinally for about 9 cm, infiltrating the soft tissues adjacent to the distal diaphysis and the metaphysis of the femur on the left. The mass has multiple cistic-like areas compatible with signs of intralesional necrosis and determines colliquative loosening and dislocation of the periosteal membrane that appears broadly interrupted. No significant erosion of the cortical bone and cancellous bone adjacent to where we except a focal thinning of the cortex. No injury to the remaining of femoral bone. The muscular structures, in particular the vastus medialis muscle and the biceps femoris, have altered perilesional signal intensity and appear displaced and compressed, without a safe plane of cleavage with the injury. Figure 1b: almost complete resolution of disease, particularly in the middle third of the distal femur; mild edema of the soft tissues and muscular fibers (arrow).

A 3-years-old child, born in Ghana and migrated to Italy one year ago, presented to our Pediatric Emergency Department complaining left knee pain. The parents reported a bike accident some days before, while the past medical history was unremarkable. Specifically, the family did not live near a watery place, in Ghana nor in Italy. The family had a dog but it was never ill and it had never bitten the child. At physical examination the left knee appeared swollen, warm and painful. The remainder of the examination was normal. The X-ray of the leg showed an extensive erosion of the cortical bone of the distal third of the diaphysis of the femur, extended cranial to caudal for about 4.5 cm, associated with periosteal reaction and opacity in the density of the soft parts on the rear edge. MRI was performed and it showed a voluminous expansive mass, extending longitudinally for about 9 cm, infiltrating the soft tissues adjacent to the distal diaphysis and the metaphysis of the femur on the left. Multiple cystic-like areas were compatible with signs of intralesional necrosis and determining colliquative loosening and dislocation of the periosteal membrane that appeared broadly interrupted. The muscular structures showed altered perilesional signal intensity and appeared displaced and compressed, without a safe plane of cleavage with the injury. The report was consistent with Ewing's sarcoma of bone extra-localization (Figure 1a). On the basis of this hypothesis we performed a total body CT scan which didn’t show any metastatic disease. Subsequently we decided to submit the child to surgical biopsy and during the incision of the muscle abundant purulent material was drained. In the suspect of a bacterial abscess, the lesion was “cleaned” and “sutured”, leaving drainage. Because the laboratory blood exams showed an elevated CRP (13.22 mg/dl), with normal WBC, and the child had a mild fever, i.v. ceftriaxone (50 mg/kg) was administered. After 15 days of i.v. treatment, the patient was discharged, with an oral amoxicillin + clavulanic acid, waiting for the histology. The drained material was cultured, but resulted negative for bacteria and common fungi. One week after discharge, the patient came to remove the stitches and the lesion was still draining material. The patient was readmitted in the ward, and radiologic exams showed a worsening of the bone lesions and the persistence of “purulent collection of about 4 x 2 cm” in the soft tissues.

**Figure 2:**
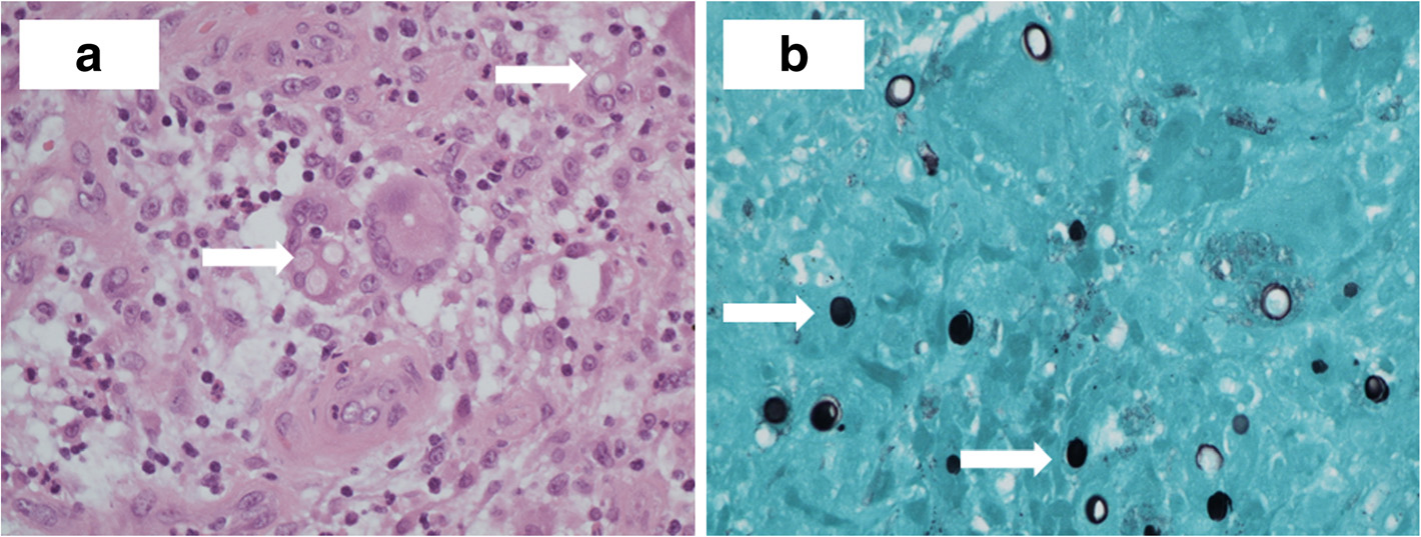
2a (haematoxylin-eosin X 400) and 2b (Gomori methenamine silver stain magnification X 400): granulomatous inflammation with the presence of giant cell formations rounded intracytoplasmic (arrows), positive for Grocott / Gomori methenamine silver stain.

Intravenous antibiotic therapy was changed and suspecting osteomyelitis we administered i.v. ceftriaxone (50 mg/kg) and amikacin (20 mg/kg). Causes of immunosuppression were ruled out. In the meantime the histological examination revealed a chronic granulomatous and necrotic process with the presence of scattered, rounded fungal spores with wall reinforcement into the cytoplasm of multinucleated giant cells (Figure 2a).

At histochemical stains, fungal structures were positive for Periodic-acid-Schiff (PAS) and methenamine silver (Grocott) (Image 2b), but negative with mucicarmine, then resulting more consistent with Blastomycosis.

The drained material newly cultured resulted again negative for bacteria and fungi and serum antibodies for Bd, C. neoformans, B. Burgdorferi; B. henselae; antigens of Aspergillus, Weil-Felix and Widal-Wright reactions, Quantiferon-TB gold resulted negative. Unfortunately in our molecular biology lab the specific primers for Blastomyces were not available at this time. Subsequently, the paucity of specimen remained did not consent to perform a valuable test with PCR. After surgical curettage, i.v. lipid amphotericin B (ampB) (0.7 mg/kg/day) was administered for 2 weeks, with evident clinical improvement. The patient was discharged with step-down therapy with oral itraconazole (10 mg/kg/day) for 6 months. Liver function, checked for possible epatotoxicity, was found normal. At 3, 9, 12 and 18-moths follow-up no limitation of movements nor pain were noted and X-Ray and MR (Figure 1b) showed total resolution of the lesion with bone reapposition.

## Conclusions

Blastomycosis is an uncommon mycotic infection, endemic in North America, but substantially unknown in Europe. This mycotic disease is rare among children and adolescents, representing only 3-10% of the cases [3]. In children, the lungs are most frequently involved. Extrapulmonary or disseminated blastomycosis occurs in approximately 10% of children and is usually heralded by prior pulmonary symptoms [4]. A review of known cases diagnosed in Africa between 1951 and 1987 revealed that, among the 81 African patients, bone involvement, with or without extension to the overlying subcutaneous tissues and skin, was the most common presentation of disease [2].

Bone is the third most common site of blastomycotic lesions, after lung and skin. Bones may be involved in 14-60% of cases of blastomycosis, and any bone can be implicated [5,6]. Synovial joint involvement has also been reported. Patients with blastomycotic osteomyelitis have pain and swelling at the site, often associated with overlying skin abscess or ulcer. Areas of bone involvement usually appear as lucent (X-ray) or low-attenuation (CT) lesions with indistinct margins but have no radiologic specific features to help distinguish them from other forms of osteomyelitis or neoplasm. Specifically, the lack of periosteal reaction radiographically diverts the differential diagnosis away from osteomyelitis [7]. Most bone lesions will resolve with antifungal treatment, but in many instances surgical treatment may be necessary, including debridement and curettage [6,8,9]. Children are allegedly unusual host for blastomycosis. As it can mimic other diseases, such as bacterial infection or malignancy, diagnosis can be mistaken or delayed even in endemic Countries. A case occurring in a non-endemic area can represent a Bd transferred from an endemic area, or a reactivation after the patient has moved from an endemic area, as it probably was in our case. Bd grows in a mycelia form at room temperature and it converts to a yeast form within tissues and in culture at 37°C. The rarity of this infection at our latitude justifies the unavailability of specific tests. Culturing the fungus in specific Sabouraud dextrose agar is highly reliable but it requires 2–6 weeks.

Antibodies to Bd detection tests are considered inadequate for diagnosis.

Antigen detection in urine, cerebrospinal fluid (CSF), bronchoalveolar lavage (BAL) fluid, serum and other sterile body fluids has low specificity, being also positive in patients with histoplasmosis and paracoccidoidiomycosis. Methods based on PCR have shown promise but they are not widely available yet [1,10]. Direct visualization of single broad-based budding yeast, stained with haematoxylin-eosin, PAS and Gomori methenamine silver (GMS) stain in sputum or tissue samples at microscopy is the primary method with which a diagnosis is made. Bd is not colored by mucicarmine stain, unlike *Cryptococcus*. As serologic search for antibodies is inadequate for diagnosis and a negative culture does not exclude the possibility of infection, the role of cytology and histopathology was determinant in our case, as already elsewhere described [9,10]. Obviously in the absence of PCR and/or DNA-RNA sequences the diagnosis of blastomycosis is only presumptive. A retrospective study of 2007 [6], reviewing 45 cases of skeletal blastomycosis, reported a median delay in diagnosis of more than 2 months. The delay in diagnosis of the present case was 30 days. In a country “destination of immigration”, Blastomycosis represents a challenge for European paediatricians, and it should be included in the differential diagnosis of unexplained infections in patients coming from endemic areas.

### Consent

Written informed consent was obtained from the patient’s parents for publication of this case report and any accompanying images. A copy of the written consent is available for review by the Series Editor of this journal.

## Abbreviations

Bd: B. dermatitidis; HE: Hematoxylin and eosin; PAS: Periodic acid-schiff; GMS: Gomori methenamine silver; CSF: Cerebrospinal fluid; BAL: Bronchoalveolar lavage; ampB: Amphotericin B.

## Competing interests

All authors declare that they have no competing interest.

## Authors**’** contributions

All authors contributed to conception and design, acquisition of data, analysis or interpretation of data and gave final approval of the version to be published. In detail: MC: reviewed the literature; MC, LI and AG conceived of the study, participated in its design and helped to draft the manuscript; LI revised the manuscript; GR: provided histological examination and images; PP: have been involved in revising the manuscript critically for important intellectual content.
